# NLRP3 Upregulation in Retinal Pigment Epithelium in Age-Related Macular Degeneration

**DOI:** 10.3390/ijms17010073

**Published:** 2016-01-08

**Authors:** Yujuan Wang, Jakub W. Hanus, Mones S. Abu-Asab, Defen Shen, Alexander Ogilvy, Jingxing Ou, Xi K. Chu, Guangpu Shi, Wei Li, Shusheng Wang, Chi-Chao Chan

**Affiliations:** 1 Immunopathology Section, Laboratory of Immunology, National Eye Institute, National Institutes of Health, Bethesda, MD 20892, USA; yujuanwang2013@gmail.com (Y.W.); defen.shen@gmail.com (D.S.); xi.kathy.chu@gmail.com (X.K.C.); 2 State Key Laboratory of Ophthalmology, Zhongshan Ophthalmic Center, Sun Yat-sen University, Guangzhou 510060, China; 3 Department of Cell and Molecular Biology, Tulane University, New Orleans, LA 70118, USA; jhanus@tulane.edu (J.W.H.); swang1@tulane.edu (S.W.); 4 Histopathology Core, National Eye Institute, National Institutes of Health, Bethesda, MD 20892, USA; mones@nei.nih.gov (M.S.A.-A.); ogilvy.alexander@gmail.com (A.O.); 5 Unit on Retinal Neurophysiology, National Eye Institute, National Institutes of Health, Bethesda, MD 20892, USA; ouj@nei.nih.gov (J.O.); liwei2@nei.nih.gov (W.L.); 6 Experimental Immunology Section, Laboratory of Immunology, National Eye Institute, National Institutes of Health, Bethesda, MD 20892, USA; shig@nei.nih.gov

**Keywords:** retina, oxidative stress, inflammation, autophagy, mitochondria

## Abstract

Inflammation and oxidative stress are involved in age-related macular degeneration (AMD) and possibly associated with an activation of neuronal apoptosis inhibitor protein/class II transcription activator of the Major Histocompatibility Complex (MHC)/heterokaryon incompatibility/telomerase-associated protein 1, leucine-rich repeat or nucleotide-binding domain, leucine-rich repeat-containing family, and pyrin domain-containing 3 (NLRP3) inflammasome. In the present study, we used a translational approach to address this hypothesis. In patients with AMD, we observed increased mRNA levels of *NLRP3*, *pro-interleukin-1 beta* (*IL-1β*) and pro-*IL-18* in AMD lesions of the retinal pigment epithelium (RPE) and photoreceptor. *In vitro*, a similar increase was evoked by oxidative stress or lipopolysaccharide (LPS) stimulation in the adult retinal pigment epithelium (ARPE-19) cell line, and the increase was reduced in siRNA transfected cells to knockdown NLRP3. Ultrastructural studies of ARPE-19 cells showed a swelling of the cytoplasm, mitochondrial damage, and occurrence of autophagosome-like structures. NLRP3 positive dots were detected within autophagosome-like structures or in the extracellular space. Next, we used a mouse model of AMD, *Ccl2/Cx3cr1* double knockout on *rd8* background (DKO *rd8*) to ascertain the *in vivo* relevance. Ultrastructural studies of the RPE of these mice showed damaged mitochondria, autophagosome-like structures, and cytoplasmic vacuoles, which are reminiscent of the pathology seen in stressed ARPE-19 cells. The data suggest that the NLRP3 inflammasome may contribute in AMD pathogenesis.

## 1. Introduction

Age-related macular degeneration (AMD) is a neurodegenerative disorder [1] and the leading cause of irreversible central blindness worldwide in the elderly [2,3,4]. AMD pathogenesis is complex and remains poorly understood. The two main phenotypes of AMD at the end-stage are geographic atrophy (GA or “dry AMD”) and neovascular/exudative AMD (nAMD or “wet AMD”). GA AMD is characterized by degeneration and atrophy of the retinal pigment epithelium (RPE) and photoreceptors in the macula, whereas nAMD is characterized by choroidal neovascularization (CNV) and fibrovascular scarring in the macula. Because the macula is in the central retina and is responsible for the sharp central vision, the end-stage of both AMD phenotypes causes severe visual loss [5].

Several studies have linked AMD to the disturbance in homeostasis and allostasis that result in allostatic overload due to a wide range of causes, such as aging, genetic predisposition, and environmental factors [5,6,7,8,9,10,11,12]. A consequence of allostatic overload is intense parainflammation. Oxidative stress, by releasing reactive oxygen species, is another crucial trigger for AMD pathogenesis [13,14,15]. Furthermore, oxidative stress is capable of further magnifying parainflammation in AMD. Overwhelming or sustained parainflammation can recruit macrophages and initiate a low-grade chronic inflammation [16], which can provoke the pathology of AMD. Under these conditions, the RPE, photoreceptors, and other retinal neurons are exposed to the proinflammatory cytokines and neurotoxins released by the activated inflammatory components. Thus, all of these factors eventually contribute to the initiation and progression of neuroretinal and RPE cell death leading to AMD [12].

Recent studies have indicated that the NACHT (neuronal apoptosis inhibitor protein, class 2 transcription activator of the MHC, heterokaryon incompatibility and telomerase-associated protein 1), LRP or NLR (nucleotide-binding domain, leucine-rich repeat-containing family), and PYD (pyrin domain)-containing protein 3 (NLRP3) inflammasome activation is involved in the pathogenesis of GA and nAMD [9,11,17,18,19,20]. The activated NLRP3 inflammasome autocatalytically cleaves caspase-1 precursor, which subsequently leads to maturation of the proinflammatory cytokines interleukin-1 beta (IL-1β) and IL-18 [21]. Furthermore, NLRP3-activated proinflammatory cytokines have been reported to have different effects in the two AMD phenotypes [11,17]. Doyle *et al.* showed a protective role for NLRP3 and IL-18 in AMD progression based on a mouse model of nAMD [17], whereas Ambati’s group demonstrated the contribution of NLRP3 inflammasome and IL-18 to RPE degeneration and potential visual loss via the *Alu* DICER (an endoribonuclease or helicase with RNase motif. DICER cleaves double-stranded RNA and pre-microRNA into short double stranded RNA fragments of small interfering RNA and microRNA) pathway in GA [11,19,22]. Although the pathological impact of the NLRP3 inflammasome in innate immunity and inflammatory response has been documented in AMD patients and animal models, its relationship with RPE damage requires further resolution and elucidation.

## 2. Results and Discussion

### 2.1. Upregulation of NLRP3 Inflammasome in the Maculae of GA and nAMD

**Figure 1:**
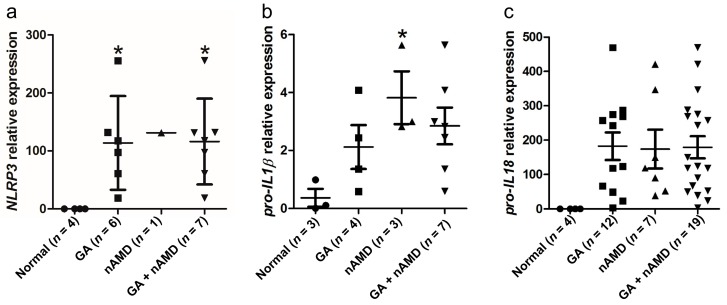
Upregulation of *NLRP3* inflammasome in the maculae of geographic atrophy (GA) and neovascular age-related macular degeneration (nAMD) patients. (**a**) *NLRP3* mRNA expression in the macular cells (mainly the photoreceptors and RPE cells) of paraffin-embedded slides of human eyes; (**b**) Pro-*IL-1β* mRNA expression in the macular cells (mainly the photoreceptors and RPE cells) of paraffin-embedded slides of human eyes; (**c**) Pro-*IL-18* mRNA expression in macular cells (mainly the photoreceptors and RPE cells) of paraffin-embedded slides of human eyes. Data are presented as mean ± SEM. * *p* < 0.05.

To determine NLRP3 inflammasome expression in AMD, we assessed *NLRP3*, pro-*IL-1β* and pro-*IL-18* transcript (mRNA) expressions by quantitative reverse transcription-polymerase chain reaction (qRT-PCR) in the macula (central retina) and peripheral retina of both GA and nAMD specimens *vs.* age-matched normal controls. We isolated RNA from macular lesions, mainly the photoreceptor and RPE cells that were microdissected from 19 paraffin-embedded eyes with advanced stage AMD (12 GA and 7 nAMD) and four control eyes. Each testing molecule (*NLRP3*, pro-*IL-1β* and pro-*IL-18* mRNA) was compared with *β-actin* mRNA, respectively. Six GA and six nAMD did not yield measurable results for *NLRP3* and *β-actin* mRNA; eight GA, four nAMD, and one control eye did not yield measurable results for pro-*IL-1β* and *β-actin* mRNA. Because some specimens showed no amplification of either *β-actin* or other target genes in those specimens (likely due to RNA degradation, strand breakage, or cross-linking during the long-term storage of archived paraffin blocks and slides), they were excluded from the final statistical analysis [23,24]. Macular *NLRP3* expression ranged from 113- to 131-fold higher in GA/GA + nAMD *vs.* normal controls (Figure 1a). Macular pro-*IL-1β* expression ranged from three- to four-fold higher in nAMD/GA + nAMD *vs.* normal controls (Figure 1b). Macular pro-*IL-18* ranged from 173- to 182-fold higher in all tested AMD groups *vs.* normal controls (Figure 1c). Nineteen AMD and four normal controls were also assayed in the peripheral retina; however, no expression of *NLRP3*, pro-*IL-1β* and pro-*IL-18* transcripts was detected in the tested specimens. Although pro-*IL-18* transcripts were not significantly upregulated in GA and nAMD macular lesions, *NLRP3* and pro-*IL-1β* levels are higher within AMD macular lesions when compared with the normal human macular area.

### 2.2. Activation of NLRP3 Inflammasome in Human RPE under Inflammation and Oxidative Stress

**Figure 2:**
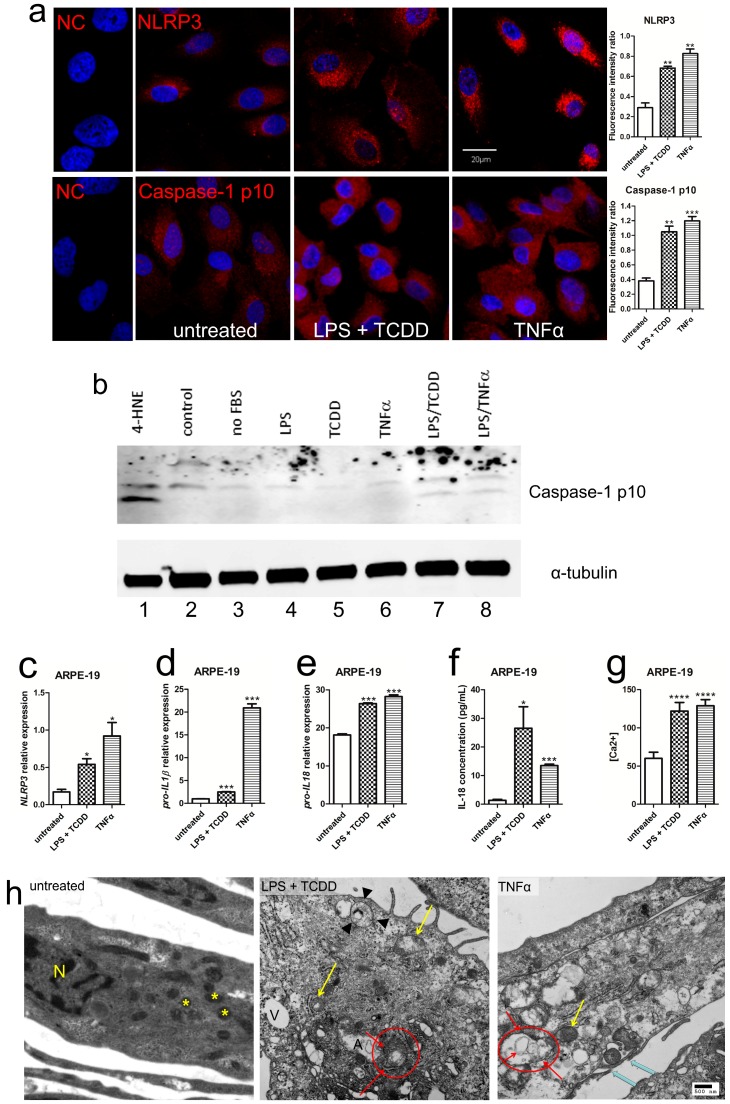

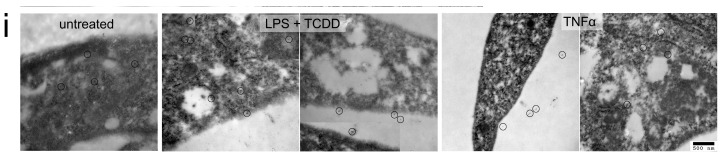
Activation of NLRP3 inflammasome in human ARPE-19 cells under inflammation and oxidative stress. (**a**) Confocal microscopy of ARPE-19 stimulated for 24 h with LPS (lipopolysaccharide) + TCDD (2,3,7,8-tetrachlorodibenzo-*p*-dioxin) and TNFα (*n* = 4). Normal IgG was used as primary antibody in the negative control (NC). NLRP3 (**upper**) and caspase-1 p10 subunit (**lower**) are labeled in **red**. The nuclei were stained with 4’,6-diamidino-2-phenylindole dihydrochloride (DAPI) (**blue**). Image-J software is used to measure the fluorescence intensity in pixels per area in each image and expressed as fluorescence intensity ratio and shows significantly higher NLRP3 and caspase-1 levels in the stimulated cells. Scale bars = 20 µm; (**b**) Western blot analyses detect higher caspase-1 p10 subunits (10 kDa) in stressed ARPE-19 cells. ARPE-19 exposed to 4-HNE (5 μg/mL) was used as a positive control for caspase-1 mediated inflammasome activation (lane **1**). Control ARPE-19 cells were cultured either in 10% FBS containing cell culture medium (lane **2**) or exposed to serum-free culture medium for 24 h (lane **3**). ARPE-19 cells were incubated in serum-free culture medium for 24 h, and subsequently treated with 10 μg/mL LPS (lane **4**), 10 nM TCDD (lane **5**), and 10 ng/mL TNFα (lane **6**). To induce inflammasome activation ARPE-19 cells were pretreated with 10 μg/mL LPS and exposed to 10 nM TCDD (lane **7**) or 10 ng/mL TNFα (lane **8**); (**c**–**e**) qRT-PCR analysis of *NLRP3*, pro-*IL-1β* and pro-*IL-18* mRNA demonstrates significantly higher levels in ARPE-19 cells stimulated with LPS + TCDD and TNFα (*n* = 4); (**f**) ELISA analysis of IL-18 measures significant increases in the supernatants of ARPE-19 cells treated with LPS + TCDD and TNFα (*n* = 4); (**g**) Ca^2+^ mobilization analysis discloses significantly higher cytosolic Ca^2+^ in ARPE-19 cells stimulated with LPS + TCDD and TNFα (*n* = 4); (**h**) Ultrastructural evaluation of ARPE-19 cells stimulated with LPS + TCDD and TNFα. Normal mitochondria (**yellow** asterisks) with distinctive membranes and cristae are seen in the untreated ARPE-19 cells (**left** panel). Damaged mitochondria show swelling, disarrangement/loss of cristae, and only outer membranes are visible (**yellow** arrows, **middle** and **right** panels). Some transitional stages from degenerative mitochondria to autophagosomes are also illustrated (arrowheads). Single-membrane autophagosomes (A) and multilayer-membrane autophagosomes are identified (**red** circles with **red** arrows indicating membranous structure, **middle** and **right** panels). The **blue** arrows point formation of a plasma-membrane pore with two opening edges. N, nucleus; A, autophagosome and autophagosome-like structure; V, vesicle. Scale bar = 500 nm; (**i**) Ultrastructural evaluation of ARPE-19 cells stimulated with LPS + TCDD and TNFα following immunolabeled for NLRP3, visualized with 15-nm gold-conjugated protein. Circles indicate NLRP3 immunolabeling positive dots that illustrate extracellular translocation. Scale bar = 500 nm. Data in (**a**) and (**c**–**g**) are presented as mean ± SEM. * *p* < 0.05; ** *p* < 0.01; *** *p* < 0.001; **** *p* < 0.0001.

In order to mimic the intraocular inflammation and oxidative stress, we used 2,3,7,8-tetrachlorodibenzo-*p*-dioxin (TCDD) and low doses of lipopolysaccharide (LPS) + TCDD as a model of oxidative stress plus low-grade inflammation, or tumor necrosis factor-alpha (TNFα) as an inflammatory stress to stimulate human RPE. Both inflammation and oxidative stress upregulated the NLRP3, cleaved caspase-1, increased IL-1β, and IL-18 protein and RNA expressions in an adult human RPE cell line named adult retinal pigment epithelium (ARPE-19) (Figure 2a–f) and adult human RPE (hRPE) cells (Figure S1a–e). Although pro-*IL-18* expression was relatively high in the control (Figure 2e), the mature IL-18 protein was very low in untreated ARPE-19 cells (Figure 2f). Moreover, an accumulation of cytosolic Ca^2+^ was recorded significantly greater when ARPE-19 cells were challenged with LPS + TCDD and TNFα (Figure 2g). This implied that mitochondrial function could be affected in these stressed cells. Ultrastructure of the stimulated ARPE-19 cells illustrated autophagosomes and/or autophagosome-like structures, mitochondrial damage, and cytoplasmic vesicles (Figure 2h); these findings are similar to the reports in human AMD pathology [25]. Similar features were also found in the stressed hRPE cells (Figure S1f). Occasionally, formation of plasma-membrane pores, which suggests pyroptosis during NLRP3 inflammasome activation, was also noted in the stressed ARPE-19 cells (Figure 2h). More importantly, transmission electron microscopy (TEM) immunolabeling showed that the NLRP3 protein either colocalized with autophagosomes/autophagosome-like structures or redistributed into the extracellular spaces under stimuli (Figure 2i). In comparison, the NLRP3 inflammasome was only observed in the cytoplasm in ARPE-19 cells without stimuli.

### 2.3. Silencing of NLRP3 Inhibits Inflammasome Activation in Human RPE Cells under Inflammation and Oxidative Stress

**Figure 3:**
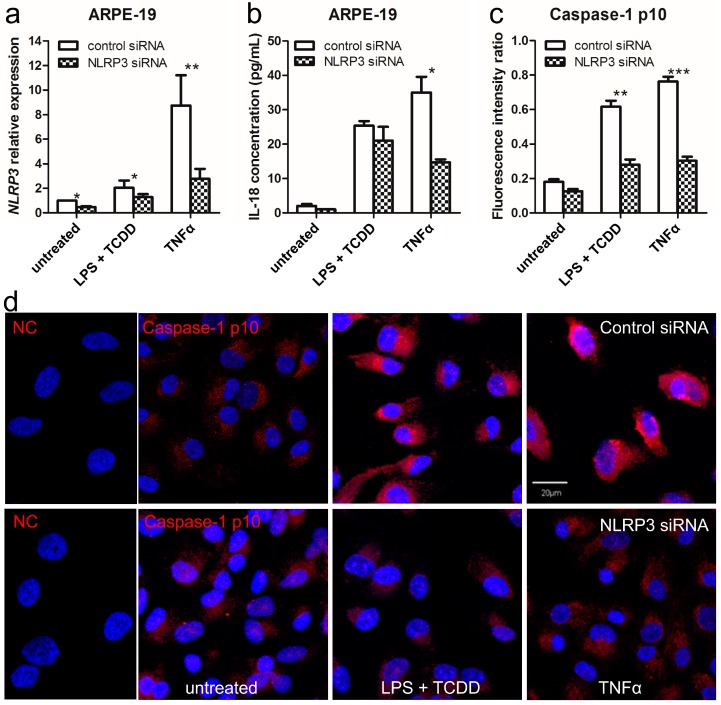
NLRP3 knockdown inhibits inflammasome activation in human ARPE-19 cells under inflammation and oxidative stress. (**a**) qRT-PCR analysis of *NLRP3* mRNA shows significantly lower in ARPE-19 cells stimulated with LPS + TCDD and TNFα for 24 h after siRNA transfection (*n* = 4); (**b**) ELISA analysis of IL-18 in the supernatants of ARPE-19 cells treated with LPS + TCDD and TNFα shows a significant decrease after siRNA transfection (*n* = 4); (**c**) The caspase-1 p10 protein ratio of immunohistochemistry is calculated. Image-J software is used to measure the band intensity in pixels (*n* = 4) and demonstrate significantly reduction in the siRNA transfected ARPE-19 cells; (**d**) Confocal microscopy of ARPE-19 treated with LPS + TCDD and TNFα for 24 h illustrates lower intensity (faint **red** color) after siRNA transfection (*n* = 4). Normal IgG was used as primary antibody in the negative control (NC). Caspase-1 p10 subunit was labeled in **red**. The nuclei were stained with DAPI (**blue**). Data are presented as mean ± SEM. * *p* < 0.05; ** *p* < 0.01; *** *p* < 0.001. Scale bar = 20 µm.

To further characterize the importance of the NLRP3 inflammasome in the pathogenesis of AMD, *NLRP3* was knocked down with small interfering RNA (siRNA) in human RPE cells prior to LPS + TCDD and TNFα stimulation (Figure 3a and Figure S2a). *NLRP3* siRNA reduced secretion of IL-1β (Figure S2b) and IL-18 (Figure 3b) as well as caspase-1 cleavage (Figure 3c and Figure S2c,d) under stimuli *vs.* control siRNA in both ARPE-19 (Figure 3) and hRPE cells (Figure S2). These data support the indispensable role of NLRP3 inflammasome in the cleaving of caspase-1 and ongoing maturation of proinflammatory IL-1β and IL-18 cytokines.

### 2.4. Upregulation of NLRP3 Inflammasome in Ccl2/Cx3cr1 double knockout on C57BL/6N (Crb1 rd8) (DKO rd8) Mouse Retina

**Figure 4:**
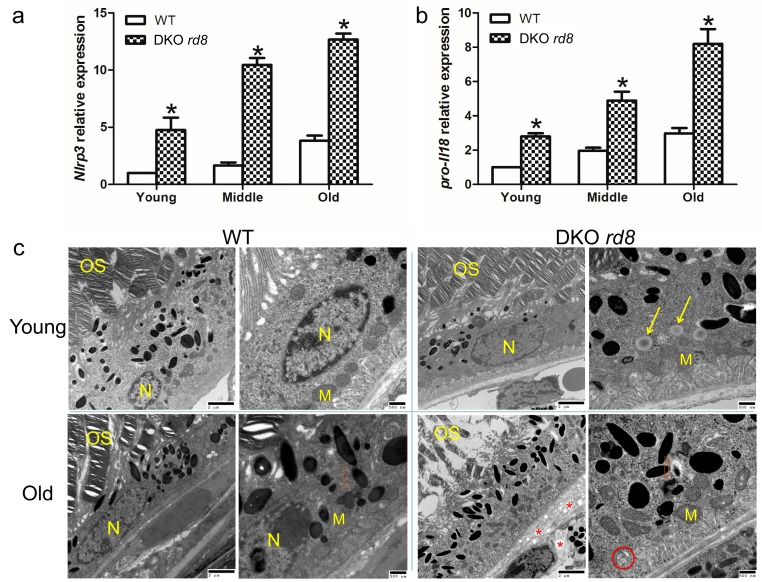
Upregulation of NLRP3 inflammasome in an AMD model of *Ccl2/Cx3cr1* double knockout on C57BL/6N (DKO *rd8*) mouse retina. (**a**) qRT-PCR analysis of *Nlrp3* mRNA in WT (C57BL/6N) and DKO *rd8* mouse retina of different age groups as young (1 month), middle (4–5 months) and old (11–12 months) (*n* = 4). Significantly higher mRNA levels are measured in mutant mice and are age-dependent; (**b**) qRT-PCR analysis of pro-*Il18* mRNA in DKO *rd8* mouse retina of different age groups (*n* = 4). Significantly higher mRNA levels are measured in mutant mice and are age-dependent; (**c**) TEM images of the retinas of young and old WT and DKO *rd8*. The young RPE cells of WT mouse show normal nuclei (N) and healthy mitochondria (M) in the cytoplasmic base. The photoreceptor outer segments (OS) are well aligned. There is a lysosome with lipid bodies (**orange** open arrow) in the RPE of old WT. In contrast, there are more lipid droplets (**yellow** arrows) in a young DKO *rd8* RPE. Lysosomes with lipid bodies (open arrow) are identified in an old DKO *rd8* RPE. Additionally, DKO *rd8* RPE showed autophagosome (**red** circle), as well as a higher number of cytoplasmic vacuoles (asterisks). The photoreceptor OS are poorly aligned and undergo degeneration. Data are presented as mean ± SEM. * *p* < 0.05. Scale bars are 2 µm and 500 nm.

Our *in vitro* results presented above revealed the capability of LPS + TCDD and TNFα to induce IL-1β and IL-18 release via activation of the NLRP3 inflammasome. To further explore the involvement of NLRP3 inflammasome in AMD, we subsequently examined the NLRP3 inflammasome pathway *in vivo* using a murine model of progressive, focal retinal degeneration that mimics certain features of human AMD including elevation of ocular A2E (a lipofuscin fluorophore) levels [26,27,28,29,30]. This model is *Ccl2/Cx3cr1* double knockout mouse on C57BL/6N background (DKO *rd8*). Additionally, like human AMD, both inflammation and oxidative stress are involved in the pathogenesis of DKO *rd8* mice, having increased expression of anti-retinal autoantibodies, greater macrophage infiltration, complement factor deposition and higher nicotinamide adenine dinucleotide phosphate/its reduced form (NADP^+^/NADPH) ratio [28,31]. Several anti-inflammatory and anti-oxidative molecules are effective to suppress pathological changes and improve retinal structure in DKO *rd8* mice [31,32,33,34]. Our previous study reported elevated pro-*Il1β* in the DKO *rd8* mouse retina as compared to wild type (WT, C57BL/6N (*Crb1 rd8*)) retina [33]. In the current study, we further compared *Nlrp3* and pro-*Il18* expressions in WT and DKO *rd8* mouse retina of different age groups. Both *Nlrp3* and pro-*Il18* transcripts were upregulated in DKO *rd8* mouse retina *vs.* WT across all age groups (Figure 4a,b). Moreover, *Nlrp3* and pro-*Il18* expressions tended to increase with aging of the WT and DKO *rd8* mice (Figure 4a,b). Transmission electron microscopy (TEM) also illustrated abnormal mitochondrial cristae and inner membranes, autophagosomes, and a higher number of cytoplasmic vacuoles in DKO *rd8* RPE *vs.* age-matched WT RPE (Figure 4c). Both old WT and DKO *rd8* mice showed lipid accumulation in RPE cells (Figure 4c). These data further indicate the intimate relationship between damaged mitochondria, NLRP3 inflammasome upregulation, and induction of autophagy in RPE cells under sustained inflammation and oxidative stress.

### 2.5. Discussion

Our data suggest that NLRP3 inflammasome upregulation is involved in AMD pathogenesis, which is consistent with the previous reports in the literature [11,18]. Additionally, we have shown that both exogenous inflammatory stimuli and oxidative stress can trigger NLRP3 inflammasome in human RPE cells via caspase-1 activation; mitochondrial damage with cytoplasmic Ca^2+^ accumulation could simultaneously occur in these stressed cells. Furthermore, we have shown the coexistence of NLRP3 protein with autophagosomes/autophagosome-like structures in the cytoplasm, implying that autophagy may play a role in RPE response and survival under inflammation or oxidative stress. The activation of the NLRP3 inflammasome is also identified in the DKO *rd8* mouse retina with focal retinal degenerative lesions mimicking AMD.

In the human AMD specimens, the nucleic acid materials in some archived human specimens were degraded due to undetectable results of both internal control and target genes. We could only analyze the limited specimens with *NLRP3* transcript expression. Based on these data, we have shown higher *NLRP3* expression in the macular areas of both GA and nAMD. However, mRNA expression levels between GA and nAMD macula were similar. This is consistent with other reports in human AMD specimens [11,18]. *IL-18* transcript only showed the tendency to be upregulated in both GA and nAMD specimens, which could be due to the limited number of human samples. Thus, analyses with large numbers of human specimens are needed to determine the involvement of NLRP3 inflammasome in different types or stages of AMD. Several independent research groups have shown the role of NLRP3 inflammasome activation in AMD pathogenesis [11,18]. Recently, increased serum IL-18 levels are reported in the GA patients than those in control subjects [35]. Although IL-18 did not have pro- or anti-angiogenic effect on laser-induced CNV mouse model in one study [35], both NLRP3 and IL-18 were reported to be protective in CNV development in another study of nAMD mouse model by Doyle *et al.* [17]. Moreover, Marneros has found that targeting NLRP3 or IL-1β does not prevent vascular endothelial growth factor A (VEGFA) induced AMD pathologies, whereas IL-18 deficiency promotes CNV lesion formation in VEGFA^hyper^ mouse choroidal flat mounts [20]. In comparison, the report by Tseng *et al.* [18] and the current study showed comparable transcript expression levels between the GA and nAMD macula. The controversial data could be affected by other factors that might interact with the NLRP3 inflammasome and/or its downstream pathways in a manner related to different ocular cells (e.g., photoreceptor, RPE, and vascular endothelium), AMD stages, as well as AMD phenotypes and genetic background. Apparently, Doyle *et al.* demonstrated the protective role of IL-18 is via regulating CNV formation in mice, which pointed the effects of IL-18 on the cells forming CNV [17,36]. However, our study focused on the pathological involvement of NLRP3 activation in RPE cells and mainly in the situation mimicking dry AMD. Due to the important role of NLRP3 inflammasome pathway in the pathogenesis of AMD, blockage of NLRP3 inflammasome is regarded as a novel and promising therapy. Recently, Fowler *et al.* found that nucleoside reverse transcriptase inhibitors (NRTIs) could inhibit P2X7-mediated NLRP3 inflammasome activation by preventing caspase-1 activation induced through *Alu* RNA [22]. They indicated that NRTIs could have dual therapeutic use to treat both GA and nAMD [22].

TNFα overexpression has been found in the RPE, stromal cells and neovascular membranes of AMD eyes [37]. Exogenous TNFα acts as one of the major inflammatory stimuli presented in nAMD. Likewise, the complications of DNA damage caused by TCDD represent chronic oxidative stress in humans. By simulating both inflammation and oxidative stress, we have shown that NLRP3 inflammasome is a key complex to be involved in human RPE. As the degenerative changes of RPE are likely to occur prior to or during the photoreceptor loss in AMD, the cytokines released by the damaged RPE would further induce photoreceptor degeneration, resulting in AMD advancement. *NLRP3* silencing in human RPE greatly decreased caspase-1 activation and the release of inflammatory IL-1β and IL-18, which promotes production of other proinflammatory cytokines. Thus, early intervention disrupting NLRP3 inflammasome might be potentially effective to hinder early geographic atrophic AMD. However, in nAMD, other factors such as VEGF need to be considered rather than isolated targeting of the NLRP3 inflammasome alone. Recently a negative correlation between VEGF and IL-18 via their effects on claudin 5, a vascular endothelial tight junction protein, was demonstrated in retinal vascular diseases in patients and animal models [38].

Cells at basal state maintain low levels of cytoplasmic Ca^2+^ so that the signal-dependent influx of Ca^2+^ leads to a rapid increase in cytoplasmic Ca^2+^ levels and induction of subsequent cellular responses. As for Ca^2+^ signaling regulation, mitochondria play a key role by taking up Ca^2+^ from the endoplasmic reticulum or extracellular space to release it to the cytoplasm [39,40]. Additionally, excessive or sustained mitochondrial Ca^2+^ uptake can result in mitochondrial damage [40,41,42]. However, Ca^2+^ signaling is necessary but not sufficient for mitochondrial damage [41,42]. Murakami *et al.* have indicated that only the stimuli that mobilize Ca^2+^ and cause mitochondrial damage can activate the NLRP3 inflammasome [40]. Paralleling the NLRP3 inflammasome activation in macrophages, significantly elevated levels of cytosolic Ca^2+^ concentrations were measured under either inflammation or oxidative stress in ARPE-19 cells. The elevated Ca^2+^ level might be associated with and/or independently of mitochondrial damage and dysfunction. Extracellular oxidative stress or inflammation can induce NLRP3 activation also independently of mitochondrial degeneration. Further studies are needed to understand the intimate mechanism of Ca^2+^ upregulation and mitochondrial changes under the stress.

Ultrastructural studies revealed degenerated mitochondria in human RPE cells under inflammation and oxidative stress. Interestingly, many autophagosome and/or autophagosome-like structures, some in the transitional stage from degenerative mitochondria to autophagosomes, were also observed. These findings support the induction of autophagy. Autophagy is regarded as a cellular response system for quality-control that can deliver damaged organelles from the cytoplasm to lysosomes for degradation [43]. In the current study, inflammation and oxidative stress could cause Ca^2+^ accumulation and mitochondrial damage. In turn, the NLRP3 inflammasome sensed mitochondrial dysfunction and was subsequently activated [44]. However, an intimate and complex relationship exists between NLRP3 inflammasome activation and autophagy [45]. When autophagy is induced in these stressed cells, it acts to limit NLRP3 inflammasome activation by engulfment, which aims to suppress inflammasome activity via diminishing reactive oxygen species generation.

Resting NLRP3 inflammasome localizes to the endoplasmic reticulum structure, while activated NLRP3 redistributes to the perinuclear space with endoplasmic reticulum and mitochondria cluster in the THP1 cells [44]. Colocalization of NLRP3 inside the autophagosomes suggests a possible negative-feedback loop between autophagy induction and NLRP3 inflammasome activation. That is, the inflammation and oxidative stress induce mitochondrial degeneration, which directly or indirectly leads to NLRP3 upregulation/activation and inflammatory cytokine release. Subsequently, autophagy is promoted to inhibit NLRP3 activation as well as to protect the stressed cells. Yet, this negative regulation only works in the setting of mild, limited stimulation, in which the cells could return to a basal healthy state or maintain homeostasis [45]. In the case of severe or sustained stimulation, autophagy could only temper inflammasome activation or maintain allostasis. Failure to clear the activated NLRP3 inflammasome through autophagy would lead to allostatic overload and the eventual death of the stimulated cells [12].

Moreover, degenerative mitochondria, NLRP3 upregulation and related molecules have also been confirmed in the DKO *rd8* mouse retina with progressive focal retinal degeneration. The mouse retina has a proinflammatory status mimicking human GA, with higher levels of TNFα, IL17A, inducible nitric oxide synthase and VEGFA [33,34,46,47,48]. These chronic proinflammatory cytokines would induce persistent stress to RPE cells, damage mitochondria, promote the NLRP3 inflammasome pathway, and cause cell death via apoptosis and probably pyroptosis. In addition, lipid accumulation in our study indicates the compromised RPE cells of old WT and DKO *rd8* mice. The accumulation of cellular lipid may not appear to have a deleterious impact on RPE function. However, lipid accumulation, combined with long-term oxidative stress, results in the formation of lipid products, which can ultimately induce RPE death [49,50]. These changes can further influence Bruch’s membrane and result in drusen formation, the hallmarks of AMD. Drusen and their components have been reported to activate the NLRP3 inflammasome [17,18]. All these factors, including lipid accumulation, chronic parainflammation, and oxidative stress would induce persistent stress to RPE cells, damage mitochondria, and promote the NLRP3 inflammasome pathway.

## 3. Experimental Section

### 3.1. Human Tissue

Human tissue usage was conducted according to the Declaration of Helsinki principles and approved by the National Eye Institute Institutional Review Board (92-EI-0113). Eyes were obtained from the National Eye Institute clinical center and Johns Hopkins Wilmer Eye Institute (12 eyes of GA, 7 eyes of nAMD and 4 eyes from age-matched normal donors). Donors signed consent forms from each institution and each institution’s Institutional Review Board approved the clinical protocol respectively.

### 3.2. Cells and Stimulations

A human RPE cell line (ARPE-19, ATCC, Manassas, VA, USA) was used in the experiments between passage numbers 6 and 8. Primary adult hRPE cells were harvested from an 87-year old male donor [51]. ARPE-19 and hRPE were incubated in serum-free culture medium for 24 h, and subsequently with the same concentrations of LPS (an endotoxin; 10 μg/mL, Sigma-Aldrich, St. Louis, MO, USA) + TCDD (a stimulant of oxidative stress; 10 nM, Sigma-Aldrich) or TNFα (a potent proinflammatory cytokine; 10 ng/mL, R & D Systems, Minneapolis, MN, USA) for 24 h.

### 3.3. Mice

The DKO *rd8* mice were generated as a model of progressive, focal retinal degeneration that mimics certain features of human AMD lesions [26,27,28,29,30]. The DKO *rd8* mice and age-matched WT mice were bred in-house. In addition to the *Ccl2/Cx3cr1* double knockout, the C57BL/6N mouse line has the *crumbs-like 1* (*Crb1*) mutation in homozygous form [27]. A single base deletion in the *Crb1* gene is known in rd8 mouse. When compared with WT mice having the same *Crb1* mutation leading to focal retinal dysplasia or dystrophy, DKO *rd8* mice have additional retinal *phenotypes* of AMD-like lesions, elevated A2E levels, increased expression of anti-retinal autoantibodies, greater macrophage infiltration and complement factor deposition, as well as RPE degeneration [28]. Additionally, ultrastructural investigation showed autophagosomes and more mitochondrial degeneration in DKO *rd8* than WT RPE [52]. The DKO *rd8* mouse strain also has earlier onset and higher penetrance than *Ccl2* and *Cx3cr1* single knockout strains [28,30]. Because this model recapitulates many of the key morphological, biochemical, and immunological characteristics of AMD, DKO *rd8* was used in this study [29,53]. All animal experiments were performed under protocols approved by the National Eye Institute’s Institutional Animal Care and Use Committee and were in compliance with the Association for Research in Vision and Ophthalmology Statement for the Use of Animals in Ophthalmic and Vision Research (1995).

### 3.4. NLRP3 siRNA Transfection

*NLRP3* siRNA (Santa Cruz Biotechnology, Santa Cruz, CA, USA) and nonspecific control siRNA (Santa Cruz Biotechnology) were transfected into ARPE-19 and hRPE cells using transfection reagents according to the protocol of the manufacturer. Briefly, for each transfection, 40 nM siRNA was diluted into 1 mL siRNA transfection medium (Santa Cruz Biotechnology) and incubated for 6 h. The mixture was replaced with serum-free culture medium and incubated for 6, 12, or 24 h before RNA extraction and immunohistochemistry.

### 3.5. Immunohistochemistry

Cultured cells were harvested and fixed in 4% (*v*/*v*) paraformaldehyde for 15 min and washed in phosphate buffered saline (PBS). The fixed slides were blocked in ICC buffer (IHC/ICC blocking buffer is compatible with organic dye-conjugated antibodies as well as unconjugated antibodies) with 5% normal goat or rabbit serum for 30 min at 4 °C. Samples were incubated overnight with primary antibodies to the following antigens: NLRP3 (1:200, Santa Cruz Biotechnology.) and caspase-1 p10 (1:100; Santa Cruz Biotechnology, Dallas, TX, USA). After washing with ICC buffer, 4’,6-diamidino-2-phenylindole dihydrochloride (DAPI, 1:1000, Invitrogen, Eugene, OR, USA) and secondary antibodies conjugated to Alexa-555 (1:400, Invitrogen) were added and incubated at room temperature for 1 h. All the slides with immunohistochemical staining were examined under an Olympus FV1000 Confocal Scanning Microscope (Olympus America Inc., Center Valley, PA, USA). Image-J software (National Institute of Mental Health, NIH, Bethesda, MD, USA) is used to measure the fluorescence intensity in pixels per area in each image and expressed as fluorescence intensity ratio. The ratio of positive staining intensity to nucleus intensity calculated by Image-J is used for the final analysis.

### 3.6. qRT-PCR

For human AMD slides, microdissection was performed manually on uncovered, hematoxylin and eosin (H & E) stained glass slides. Approximately equal numbers of cells from the macular areas were microdissected. Total RNA was isolated from macular cells using Arcturus Paradise RNA isolation kit (Molecular Devices, Sunnyvale, CA, USA). For cultured cells, total RNA was isolated from cultured cells using an RNeasy Mini Kit (Qiagen, Hilden, Germany). Equal amounts of RNA were reverse transcribed with Superscript II RNase H Reverse Transcriptase (Invitrogen, Grand Island, NY, USA) to cDNA. qRT-PCR was performed on the resulting cDNA using Brilliant SYBR Green QPCR Master Mix (Stratagene, La Jolla, CA, USA). The comparative cycle threshold (*C*_t_) value method, representing log transformation, was used to establish relative quantification of the fold changes in gene expression using 7500 Real Time PCR System (Life Technologies Co., Carlsbad, CA, USA). *β-actin* was used as an internal control (a commonly used loading control for gene degradation in PCR). Primers of human and mouse *β-actin*, *Nlrp3*, *IL-1β*, and *IL-18* were purchased from SABiosciences (Frederick, MD, USA). For microdissected samples, we excluded the sample if the expression of both *β-actin* and the independently testing molecules (*NLRP3*, *IL-1β* or *IL-18*) was both below the detectable levels from the samples, a result consistent with degraded DNA.

### 3.7. Enzyme-Linked Immunosorbent Assay (ELISA)

Cell culture supernatants were used to measure IL-1β using ELISA kits from R & D Systems and IL-18 using ELISA kits from MBL (Woburn, MA, USA), according to the manufacturer’s instructions.

### 3.8. Western Blot

ARPE-19 cells were collected by trypsinization, washed briefly with PBS, and resuspended in lysis buffer (50 mM Tris-HCl (pH 7.4), 150 mM NaCl, 1 mM EDTA, 1% Triton X-100) supplemented with protease and phosphatase inhibitors (Thermo Scientific, Rockford, IL, USA). Antibodies used included: rabbit anti-caspase-1 p10 (1:500; Santa Cruz Biotechnology, Dallas, TX, USA) and mouse monoclonal anti-α-Tubulin (1:1000, Cell Signaling, Danvers, MA, USA). Following primary antibody incubation, membranes were probed with IRDye 800CW donkey-anti-mouse IgG (LiCOR) or IRDye 680RD goat-anti-rabbit IgG (LiCOR) secondary antibodies, and imaged and quantified using the LiCOR Odyssey system (LI-COR Biosciences, Lincoln, NE, USA).

### 3.9. Transmission Electron Microscopy (TEM)

ARPE-19 and hRPE cells were double-fixed in 2.5% gluteraldehyde and osmium tetroxide (0.5%), dehydrated, and embedded in Spurr’s epoxy resin. Ultrathin sections (90 nm) were prepared and double-stained with uranyl acetate and lead citrate. For immunolabeling, the cells were fixed in 5% buffered formalin and pelleted in 1.5% (*w*t/*v*) low-melting-point agarose, washed with PBS, dehydrated and embedded in London Resin (LR) White. Ultrathin sections were mounted on 150-mesh uncoated nickel grids. Grids were floated for 20 min on blocking solution (0.1% (*v*/*v*) Tween-20 and 0.5% (*w*t/*v*) cold-water fish gelatin (Ted Pella) in PBS), incubated for 1 h with anti-NLRP3 antibody, rinsed 3 times, incubated for 1 h with 15-nm gold-conjugated protein A (Ted Pella), rinsed in PBS and then water, and air-dried. Sections were stained with aqueous uranyl acetate (5%) and viewed with a JEOL JEM 1010 transmission electron microscope (JEOL Ltd., Akishima, Tokyo, Japan).

### 3.10. Ca^2+^ Imaging

ARPE-19 cells were cultured on coverslips to 50%–70% confluence. After serum-starvation and treatments, the cover slips were washed briefly with serum free culture medium. Then the cells were incubated in the same medium with 2.5 μM Fluo-4-AM dye (Invitrogen) at 37 °C for 30 min. Excessive dye was then rinsed using a 3 mM calcium buffer, and time-lapse calcium imaging was performed and recorded on a LSM510 confocal microscope with continuous irrigation. A few cells in-focus were selected, and when the fluorescent intensity stabilized, the irrigating buffer was switched to 0 mM calcium buffer containing 5 μM Ionomycin (Sigma-Aldrich). When the fluorescent signals gradually decreased and stabilized, the irrigating buffer was switched to 20 mM calcium buffer with 5 μM Ionomycin until the fluorescent signals reached maximum. The average intensities of fluorescence at 3, 0 and 20 mM calcium buffer (F_3mM_, F_0mM_ and F_20mM_) was obtained, and the intracellular calcium concentration [Ca^2+^]i (nM) was calculated as [Ca^2+^]i = 345 × (F_3mM_ − F_0mM_)/(F_20mM_ − F_0mM_) [54].

### 3.11. Statistical Analysis

GraphPad was used for statistical tests. Data are presented as mean ± SEM unless stated otherwise. In human samples, except for not comparing the *NLRP3* transcript expression in normal and nAMD (only samples in the nAMD group yield a measurable result), a one-way analysis of variance (ANOVA) test and Dunnett’s test were performed to analyze the human AMD specimens. Unpaired Student’s *t* tests were performed to compare data between control and stressed cell groups in experiments *in vitro*. In the mouse experiments, unpaired Student’s *t* tests were performed to compare between WT and DKO *rd8* mice. *p* values <0.05 were considered significant.

## 4. Conclusions

This study demonstrates a functional role for NLRP3 inflammasome pathway involvement in AMD development. Under sustained and chronic inflammation and oxidative stress, RPE cells are injured due to mitochondrial disintegration and NLRP3 inflammasome activation, causing subsequent release of IL-1β and IL-18 and leading to other proinflammatory responses that cause cell death. These findings are highly associated with AMD pathogenesis and progression.

## Supplementary Materials

Supplementary materials can be found at http://www.mdpi.com/1422-0067/17/1/73/s1.
