# Supplementary material for: NLRP3 Upregulation in Retinal Pigment Epithelium in Age-Related Macular Degeneration
Source: Int J Mol Sci. 2016 Jan 8;17(1):73. doi: 10.3390/ijms17010073 (PMC4730317; doi:10.3390/ijms17010073)
Supplement: Supplementary file 1 [file ijms-17-00073-s001.pdf]

Supplementary Materials: NLRP3 Upregulation in Retinal Pigment Epithelium in Age-Related Macular Degeneration

Yujuan Wang, Jakub W. Hanus, Mones S. Abu-Asab, Defen Shen, Alexander Ogilvy, Jingxing Ou, Xi K. Chu, Guangpu Shi, Wei Li, Shusheng Wang and Chi-Chao Chan

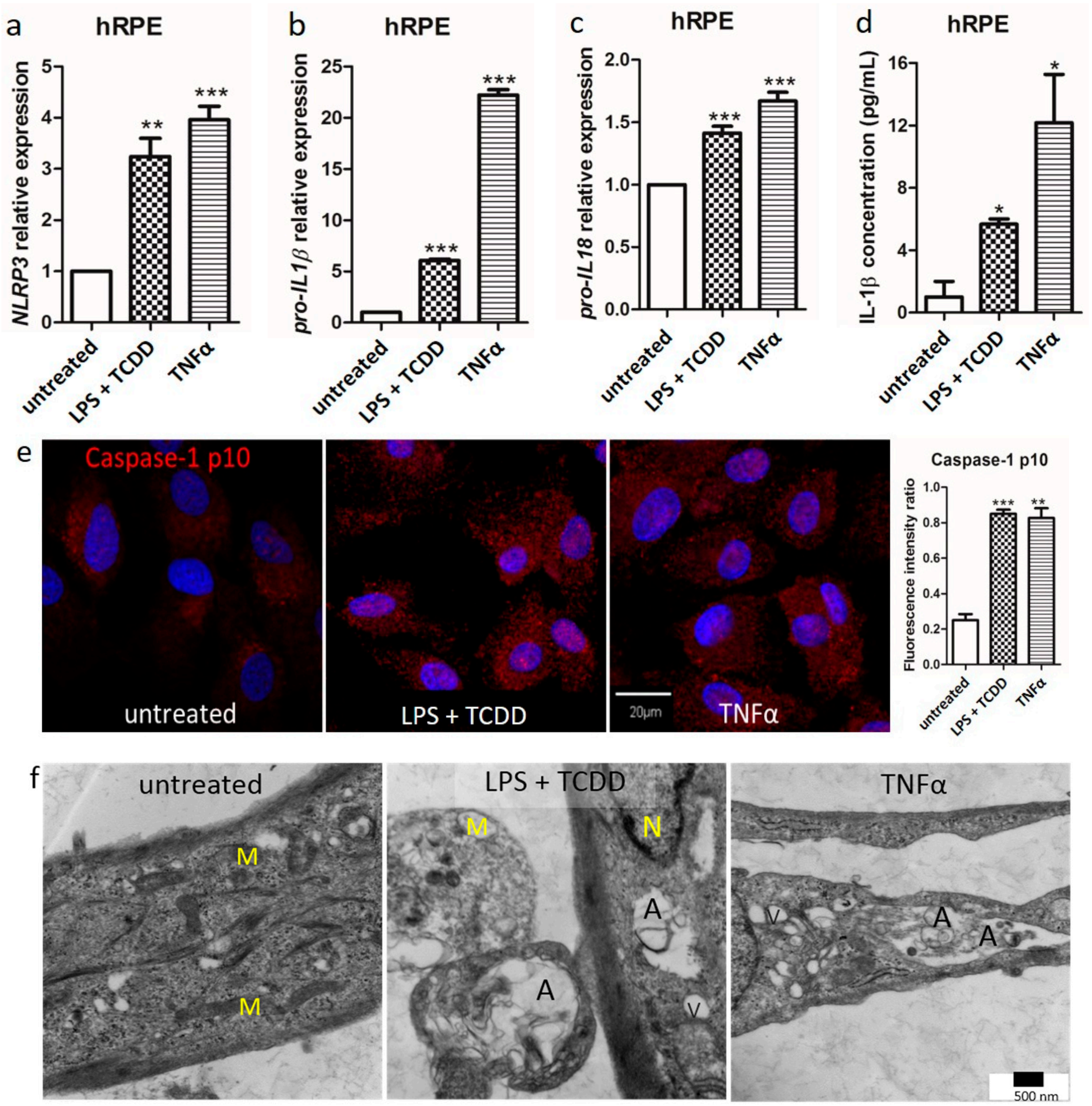

Figure S1. Activation of NLRP3 inflammasome in primary adult human RPE (hRPE) cells under inflammation and oxidative stress. (**a**–**c**) qRT-PCR analysis of *NLRP3*, pro-*IL1β* and pro-*IL18* in hRPE cells stimulated with LPS + TCDD and TNF*α* ($n = 4$); (**d**) ELISA analysis of IL-1β in the supernatants of hRPE cells treated with LPS + TCDD and TNF*α* ($n = 4$); (**e**) Confocal microscopy of hRPE stimulated for 24 h with LPS + TCDD and TNF*α* ($n = 4$). Caspase-1 p10 subunit was labeled in red. The nuclei were stained with 4,6-diamidino-2-phenylindole dihydrochloride (DAPI) (blue). Image-J software is used to measure the fluorescence intensity in pixels per area in each image and expressed as fluorescence intensity ratio. Scale bar = 20 μm; (**f**) Ultrastructural evaluation of hRPE cells stimulated with LPS + TCDD and TNF*α*. Scale bar = 500 nm. N, nuclear; M, mitochondrion; A, autophagosome; V, vesicle. Data are presented as mean ± SEM. * $p < 0.05$, ** $p < 0.01$, *** $p < 0.001$.

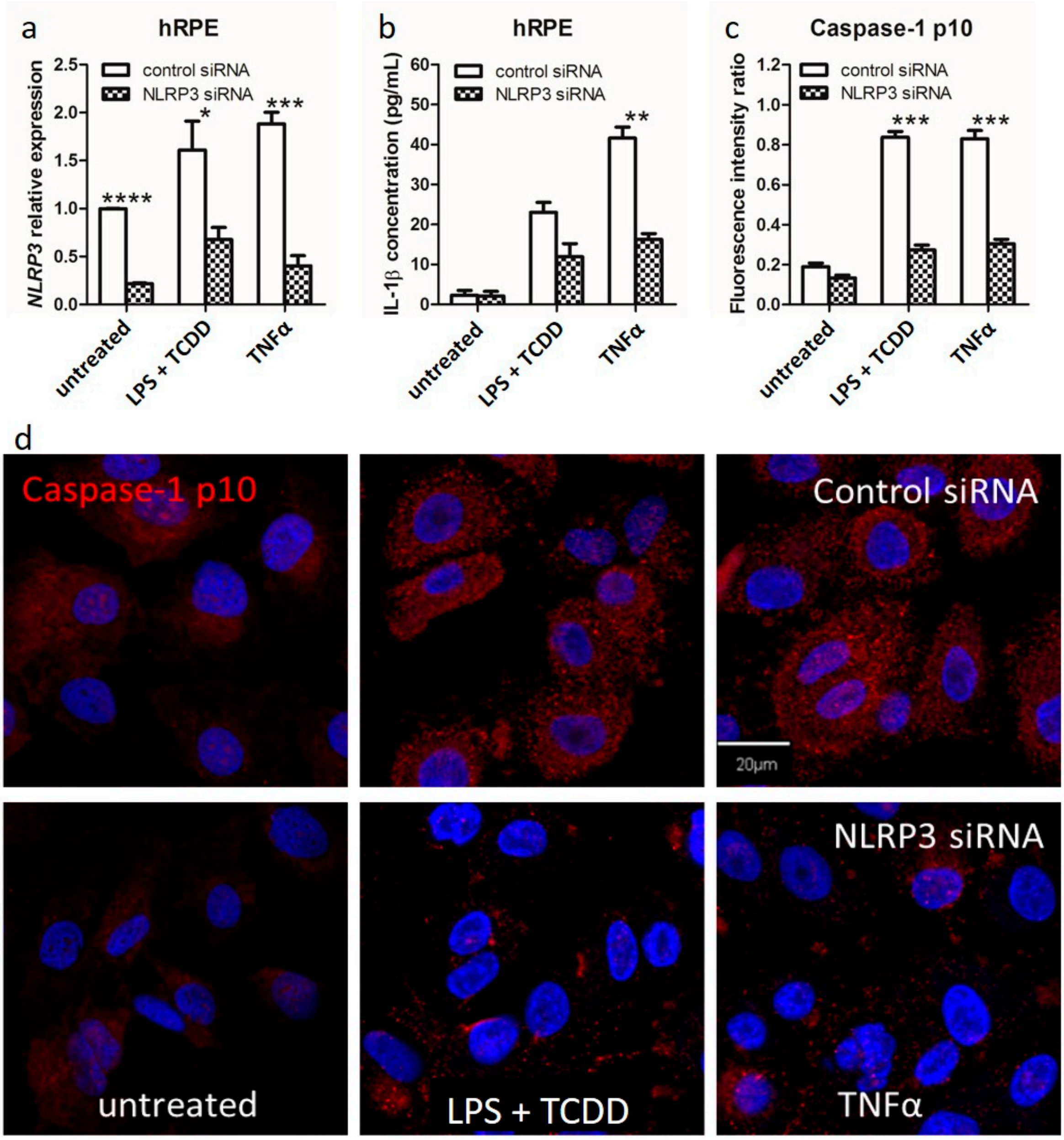

Figure S2. NLRP3 knockdown inhibits inflammasome activation in hRPE cells under inflammation and oxidative stress. (**a**) qRT-PCR analysis of *NLRP3* in hRPE cells stimulated with LPS + TCDD and TNF*α* for 24 h after siRNA transfection ($n = 4$); (**b**) ELISA analysis of IL-1β in the supernatants of hRPE cells treated with LPS + TCDD and TNF*α* after siRNA transfection ($n = 4$); (**c**) The Caspase-1 p10 protein ratio of immunohistochemistry is calculated. Image-J software is used to measure the band intensity in pixels ($n = 4$); (**d**) Confocal microscopy of hRPE cells treated with LPS + TCDD and TNF*α* for 24 h after siRNA transfection ($n = 4$). Caspase-1 p10 subunit was labeled in red. The nuclei were stained with DAPI (blue). Scale bar = 20 μm. Data are presented as mean ± SEM. * $p < 0.05$, ** $p < 0.01$, *** $p < 0.001$, **** $p < 0.0001$.
